# Molecular-based detection of the gastrointestinal pathogen *Campylobacter ureolyticus* in unpasteurized milk samples from two cattle farms in Ireland

**DOI:** 10.1186/1757-4749-4-14

**Published:** 2012-11-14

**Authors:** Monika Koziel, Brigid Lucey, Susan Bullman, Gerard D Corcoran, Roy D Sleator

**Affiliations:** 1 Department of Biological Sciences, Cork Institute of Technology, Rossa Avenue, Bishopstown, Cork, Ireland; 2 Department of Microbiology, Cork University Hospital, Wilton, Cork, Ireland

**Keywords:** Campylobacter, Emerging pathogen, Food chain, Reservoir, Dairy

## Abstract

*Campylobacter jejuni* and *coli* are collectively regarded as the most prevalent cause of bacterial foodborne illness worldwide. An emerging species, *Campylobacter ureolyticus* has recently been detected in patients with gastroenteritis, however, the source of this organism has, until now, remained unclear. Herein, we describe the molecular-based detection of this pathogen in bovine faeces (1/20) and unpasteurized milk (6/47) but not in poultry (chicken wings and caeca). This is, to the best of our knowledge, the first report of the presence of this potential gastrointestinal pathogen in an animal source, possibly suggesting a route for its transmission to humans.

## Correspondence

Human campylobacterosis is generally regarded as a zoonosis with numerous reservoirs in the natural environment [1]. While *Campylobacter* spp. rarely cause disease in livestock, foodstuffs originating from such animals appear to be the most common source of infection for humans.

Despite the fact that there are 25 species belonging to the *Campylobacter* genus [2], the primary focus of the food safety and protection agencies to date, in conjunction with clinical laboratories, has been the detection of thermophilic *Campylobacter* species associated with human diseases [3,4]. However, advances in molecular detection systems continue to highlight the fact that routine *Campylobacter* culture methods, employed by the majority of clinical laboratories, are incapable of detecting the fastidious and non-thermophilic *Campylobacter* spp [5]. Recent work in our laboratory has focused on the identification and characterisation of these atypical species of potential clinical importance [3,6]. Although failing to grow in routine culture, species specific PCR following a multiplex PCR based detection system (EntericBio, Serosep Ltd. Limerick, Ireland) identified *C. ureolyticus* in 23.8% of 349 previously genus-positive samples [6], making *C. ureolyticus* the second most common *Campylobacter* species (after *C. jejuni*) detected in faecal samples of patients presenting with gastroenteritis in Southern Ireland. Moreover, this species has also been detected and isolated from a number of patients presenting with Crohn's disease [7] and Ulcerative Colitis [8]; raising further questions as to its potential role as a significant human pathogen. Importantly, *C. ureolyticus* has been shown to be capable of attaching and translocating through the intestinal epithelia, inducing cellular damage and microvillus degradation [9].

The source of *C. ureolyticus* has, until now, remained unknown. Herein, we report for the first time the presence of this bacterium in cow’s milk, which goes some way to answering the important question as to where this emerging gastrointestinal pathogen might be coming from.

**Figure 1:**
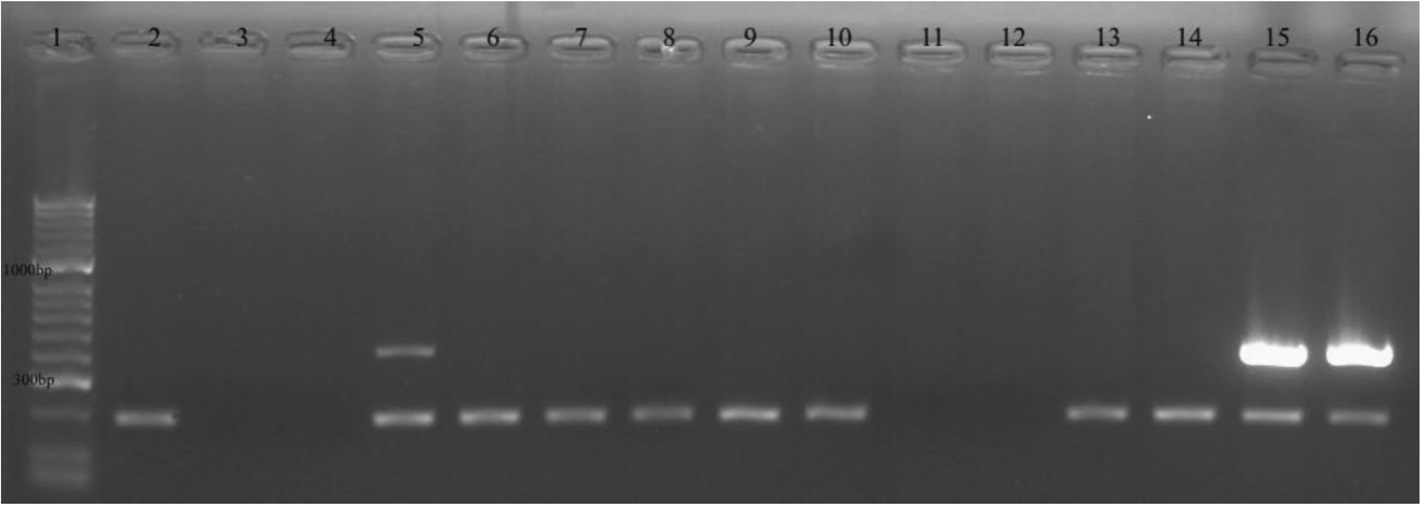
**1.5% agarose gel with PCR amplicons.** An example of a C*. ureolyticus* PCR-positive result in milk sample with a product of 429bp (Lane 5). Lane 1 100bp molecular marker; Lane 2 negative (no template) control; Lane 3–14 Milk samples; Lane 15 Extraction positive control; Lane 16 PCR positive control.

A total of 40 chicken caeca and 20 chicken wings were obtained directly from an Irish broiler processing farm between 26 January and 14 February, 2012. Bovine samples consisting of: 47 unpasteurised milk samples, 20 midstream urine samples and 20 faecal samples were also collected from individual cows between 19 April and 19 May 2012, from two Southern Irish farms, located approximately 80km apart. Bacterial DNA was extracted using QIAamp DNA Stool Mini Kit (QIAGEN) for both chicken sample types and bovine faeces and QIAamp DNA Mini Kit (QIAGEN) for bovine urine and milk samples. Each extraction run included an extraction control - a sample matrix spiked with the *C. ureolyticus* DSM 20703 strain. The presence of *C. ureolyticus* in each sample was investigated using PCR with a specific primer set targeting the *hsp60* gene of *C. ureolyticus*[6] Figure 1. An internal amplification control (IAC), designed using DNA from *N. gonorrheae* and *N. gonorrheae*-specific Pgi primer [10], was included in each reaction to assess potential PCR inhibition. The sequences of all primers used in the study are outlined in Table 1. PCR was performed using HotStarTaq DNA Polymerase (QIAGEN) with the following cycle conditions: initial denaturation for 15 min at 95°C, followed by 35 cycles of denaturation at 95°C for 30 s, annealing at 58°C for 1 min, extension at 72°C for 1 min and final extension at 72°C for 10 min.

**Table 1:** Primer sequences forward (F) and reverse (R) used in this study

**Primer**	**Primer Sequence (5'→3')**	**Product (bp)**	**Ref.**
CU-HSP60 F	GAA GTA AAA AGA GGA ATG GAT AAA GAA GC	429	[6]
CU-HSP60 R	CTT CAC CTT CAA TAT CCT CAG CAA TAA TTA AAA GA		
Pgi F	AAA CAC CTT CAC GAC TTA CCG	196	[10]
Pgi R	CCA ACT CGA ACA GTA GGG ACA		

Amplicons were visualised by electrophoresis on agarose gels and the results were interpreted on the basis of presence/absence of a band for *C. ureolyticus* positive samples and an IAC amplification for *C. ureolyticus* negative samples.

We have investigated a total of 60 chicken samples as potential reservoirs of *C. ureolyticus*. However, none gave the amplicon of the predicted size and as such were deemed negative for the presence of *C. ureolyticus*. The IAC was positive in all reactions. The fact that all of the chicken samples screened throughout the period of the study were negative for *C. ureolyticus*-specific PCR suggested that, unlike the thermophilic *C. jejuni*, chickens might not be the main reservoir for *C. ureolyticus*.

Failure to detect *C. ureolyticus* in chickens prompted us to investigate cattle, which have also been reported as a source of Campylobacter spp [11]. A total of 87 bovine samples were screened and while no *C. ureolyticus* was detected in urine samples; one faecal sample and six milk samples were positive for *C. ureolyticus* (Additional file 1). These positive samples were collected from six different cows across two geographically separate herds. To ensure the validity of the positive results, the PCR amplicons were sequenced using previously described forward and reverse *hsp60* primers. The sequences were analysed by BLAST using the NCBI database and their identity was confirmed as the *C. ureolyticus hsp60* gene.

Data reported for the prevalence of *C. ureolyticus* in patients with gastroenteritis in Ireland suggest a seasonal distribution of the organism, with the majority of cases reported in early spring, particularly during the month of March [3]. The detection of *C. ureolyticus* in bovine samples during this period suggests the need for a comprehensive prospective study to determine the incidence of this organism in cattle throughout the calendar year for a comparison with the seasonal distribution that has already been observed among patients with gastroenteritis [3]. Furthermore, comparison of MLST profiles of those strains isolated from the cattle and patients with gastrointestinal (GI) illness will significantly aid in determining if cow’s milk is in fact the true source of *C. ureolyticus* in patients with GI illness and if *C. ureolyticus*, like many of the other Campylobacter species, can be added to the growing list of zoonotic pathogens.

While more extensive studies are needed to investigate the true prevalence of the *C. ureolyticus* in cattle; the current study is, to the best of our knowledge, the first report of the presence of this bacterium in animal samples, providing a clue to a potential source of *C. ureolyticus* in the food chain.

## Competing interests

The authors declare that they have no competing interests.

## Supplementary Material

Additional file 1 Details of the samples tested in this study and their results. Click here for file
