# Supplementary material for: Molecular-based detection of the gastrointestinal pathogen Campylobacter ureolyticus in unpasteurized milk samples from two cattle farms in Ireland
Source: Gut Pathog. 2012 Nov 14;4:14. doi: 10.1186/1757-4749-4-14 (PMC3549725; doi:10.1186/1757-4749-4-14)
Supplement: Additional file 1 — Details of the samples tested in this study and their results. [file 1757-4749-4-14-S1.pdf]

Additional file 1

Details of the samples tested in this study and their results.

Sample Type	ID	Date	Result	Additional Information
Chicken Caeca	002	26/01/12	Negative	Poultry processing plant, co. Cavan
	003	26/01/12	Negative	Poultry processing plant, co. Cavan
	004	26/01/12	Negative	Poultry processing plant, co. Cavan
	005	26/01/12	Negative	Poultry processing plant, co. Cavan
	006	26/01/12	Negative	Poultry processing plant, co. Cavan
	007	26/01/12	Negative	Poultry processing plant, co. Cavan
	008	26/01/12	Negative	Poultry processing plant, co. Cavan
	009	26/01/12	Negative	Poultry processing plant, co. Cavan
	010	26/01/12	Negative	Poultry processing plant, co. Cavan
	011	03/02/12	Negative	Poultry processing plant, co. Cavan
	012	03/02/12	Negative	Poultry processing plant, co. Cavan
	013	03/02/12	Negative	Poultry processing plant, co. Cavan
	014	03/02/12	Negative	Poultry processing plant, co. Cavan
	015	03/02/12	Negative	Poultry processing plant, co. Cavan
	016	03/02/12	Negative	Poultry processing plant, co. Cavan
	017	03/02/12	Negative	Poultry processing plant, co. Cavan
	018	03/02/12	Negative	Poultry processing plant, co. Cavan
	019	03/02/12	Negative	Poultry processing plant, co. Cavan
	020	03/02/12	Negative	Poultry processing plant, co. Cavan
	021	08/02/12	Negative	Poultry processing plant, co. Cavan
	022	08/02/12	Negative	Poultry processing plant, co. Cavan
	023	08/02/12	Negative	Poultry processing plant, co. Cavan
	024	08/02/12	Negative	Poultry processing plant, co. Cavan
	025	08/02/12	Negative	Poultry processing plant, co. Cavan
	026	08/02/12	Negative	Poultry processing plant, co. Cavan
	027	08/02/12	Negative	Poultry processing plant, co. Cavan
	028	08/02/12	Negative	Poultry processing plant, co. Cavan
	029	08/02/12	Negative	Poultry processing plant, co. Cavan
	030	08/02/12	Negative	Poultry processing plant, co. Cavan
	031	08/02/12	Negative	Poultry processing plant, co. Cavan
	032	08/02/12	Negative	Poultry processing plant, co. Cavan
	033	08/02/12	Negative	Poultry processing plant, co. Cavan
	034	08/02/12	Negative	Poultry processing plant, co. Cavan
	035	08/02/12	Negative	Poultry processing plant, co. Cavan
	036	08/02/12	Negative	Poultry processing plant, co. Cavan
	037	08/02/12	Negative	Poultry processing plant, co. Cavan
	038	08/02/12	Negative	Poultry processing plant, co. Cavan
	039	08/02/12	Negative	Poultry processing plant, co. Cavan

	040	08/02/12	Negative	Poultry processing plant, co. Cavan
Chicken wings	001	14/02/12	Negative	Poultry processing plant, co. Cavan
	002	14/02/12	Negative	Poultry processing plant, co. Cavan
	003	14/02/12	Negative	Poultry processing plant, co. Cavan
	004	14/02/12	Negative	Poultry processing plant, co. Cavan
	005	14/02/12	Negative	Poultry processing plant, co. Cavan
	006	14/02/12	Negative	Poultry processing plant, co. Cavan
	007	14/02/12	Negative	Poultry processing plant, co. Cavan
	008	14/02/12	Negative	Poultry processing plant, co. Cavan
	009	14/02/12	Negative	Poultry processing plant, co. Cavan
	010	14/02/12	Negative	Poultry processing plant, co. Cavan
	011	14/02/12	Negative	Poultry processing plant, co. Cavan
	012	14/02/12	Negative	Poultry processing plant, co. Cavan
	013	14/02/12	Negative	Poultry processing plant, co. Cavan
	014	14/02/12	Negative	Poultry processing plant, co. Cavan
	015	14/02/12	Negative	Poultry processing plant, co. Cavan
	016	14/02/12	Negative	Poultry processing plant, co. Cavan
	017	14/02/12	Negative	Poultry processing plant, co. Cavan
	018	14/02/12	Negative	Poultry processing plant, co. Cavan
	019	14/02/12	Negative	Poultry processing plant, co. Cavan
	020	14/02/12	Negative	Poultry processing plant, co. Cavan
Bovine feaces	821	19/04/12	Negative	Herd 1, Southern Ireland
	867	19/04/12	Negative	Herd 1, Southern Ireland
	805	19/04/12	Negative	Herd 1, Southern Ireland
	001	20/04/12	Negative	Herd 2, Southern Ireland
	002	20/04/12	Negative	Herd 2, Southern Ireland
	003	20/04/12	Negative	Herd 2, Southern Ireland
	119	26/04/12	Negative	Herd 1, Southern Ireland
	768	26/04/12	Negative	Herd 1, Southern Ireland
	766	26/04/12	Negative	Herd 1, Southern Ireland
	673	26/04/12	Negative	Herd 1, Southern Ireland
	970[a]	26/04/12	PCR Inhibited	Herd 1, Southern Ireland
	491[a]	10/05/12	PCR Inhibited	Herd 2, Southern Ireland
	960	10/05/12	Negative	Herd 1, Southern Ireland
	713	10/05/12	Negative	Herd 1, Southern Ireland
	971	11/05/12	Negative	Herd 1, Southern Ireland
	895	11/05/12	Negative	Herd 1, Southern Ireland
	530	10/05/12	Negative	Herd 1, Southern Ireland
	840	14/05/12	Negative	Herd 1, Southern Ireland
	693	14/05/12	Negative	Herd 1, Southern Ireland
	899	14/05/12	Negative	Herd 1, Southern Ireland
	**897**	**14/05/12**	**Positive**	**Herd 1, Southern Ireland**

	779	14/05/12	Negative	Herd 1, Southern Ireland
	942	14/05/12	Negative	Herd 1, Southern Ireland
Bovine urine	887	19/04/12	Negative	Herd 1, Southern Ireland
	874	19/04/12	Negative	Herd 1, Southern Ireland
	905	19/04/12	Negative	Herd 1, Southern Ireland
	673	23/04/12	Negative	Herd 1, Southern Ireland
	604	23/04/12	Negative	Herd 1, Southern Ireland
	143	23/04/12	Negative	Herd 1, Southern Ireland
	768	02/05/12	Negative	Herd 1, Southern Ireland
	960	02/05/12	Negative	Herd 1, Southern Ireland
	889	02/05/12	Negative	Herd 1, Southern Ireland
	800	02/05/12	Negative	Herd 1, Southern Ireland
	972	02/05/12	Negative	Herd 1, Southern Ireland
	845	02/05/12	Negative	Herd 1, Southern Ireland
	806[a]	09/05/12	PCR Inhibited	Herd 1, Southern Ireland
	897	09/05/12	Negative	Herd 1, Southern Ireland
	966[a]	09/05/12	PCR Inhibited	Herd 1, Southern Ireland
	866	15/05/12	Negative	Herd 1, Southern Ireland
	530	17/05/12	Negative	Herd 1, Southern Ireland
	906	17/05/12	Negative	Herd 1, Southern Ireland
	602	17/05/12	Negative	Herd 1, Southern Ireland
	462	17/05/12	Negative	Herd 1, Southern Ireland
	731	17/05/12	Negative	Herd 1, Southern Ireland
	899	17/05/12	Negative	Herd 1, Southern Ireland
Bovine milk	887	19/04/12	Negative	Herd 1, Southern Ireland
	693	19/04/12	Negative	Herd 1, Southern Ireland
	905	19/04/12	Negative	Herd 1, Southern Ireland
	805	19/04/12	Negative	Herd 1, Southern Ireland
	771[b]	25/04/12	Negative	Herd 1, Southern Ireland
	505[b]	25/04/12	Negative	Herd 1, Southern Ireland
	178	25/04/12	Negative	Herd 1, Southern Ireland
	830	25/04/12	Negative	Herd 1, Southern Ireland
	709	25/04/12	Negative	Herd 1, Southern Ireland
	**642**	**25/04/12**	**Positive**	**Herd 1, Southern Ireland**
	**942**	**30/04/12**	**Positive**	**Herd 1, Southern Ireland**
	**344**	**30/04/12**	**Positive**	**Herd 1, Southern Ireland**
	800	30/04/12	Negative	Herd 1, Southern Ireland
	841	30/04/12	Negative	Herd 1, Southern Ireland
	596	30/04/12	Negative	Herd 1, Southern Ireland
	**960**	**30/04/12**	**Positive**	**Herd 1, Southern Ireland**
	555	30/04/12	Negative	Herd 1, Southern Ireland
	426	03/05/12	Negative	Herd 2, Southern Ireland

	415	03/05/12	Negative	Herd 2, Southern Ireland
	294	03/05/12	Negative	Herd 2, Southern Ireland
	219	03/05/12	Negative	Herd 2, Southern Ireland
	384	03/05/12	Negative	Herd 2, Southern Ireland
	162	03/05/12	Negative	Herd 2, Southern Ireland
	**491**	**03/05/12**	**Positive**	**Herd 2, Southern Ireland**
	351	03/05/12	Negative	Herd 2, Southern Ireland
	368	03/05/12	Negative	Herd 2, Southern Ireland
	341	03/05/12	Negative	Herd 2, Southern Ireland
	619	08/05/12	Negative	Herd 1, Southern Ireland
	897	08/05/12	Negative	Herd 1, Southern Ireland
	866	08/05/12	Negative	Herd 1, Southern Ireland
	576	08/05/12	Negative	Herd 1, Southern Ireland
	713	08/05/12	Negative	Herd 1, Southern Ireland
	797	08/05/12	Negative	Herd 1, Southern Ireland
	941	08/05/12	Negative	Herd 1, Southern Ireland
	311	10/05/12	Negative	Herd 2, Southern Ireland
	418	10/05/12	Negative	Herd 2, Southern Ireland
	150	10/05/12	Negative	Herd 2, Southern Ireland
	278	10/05/12	Negative	Herd 2, Southern Ireland
	369	10/05/12	Negative	Herd 2, Southern Ireland
	**216**	**10/05/12**	**Positive**	**Herd 2, Southern Ireland**
	284	10/05/12	Negative	Herd 2, Southern Ireland
	322	10/05/12	Negative	Herd 2, Southern Ireland
	899	17/05/12	Negative	Herd 1, Southern Ireland
	296	17/05/12	Negative	Herd 2, Southern Ireland
	314	17/05/12	Negative	Herd 2, Southern Ireland
	487	17/05/12	Negative	Herd 2, Southern Ireland

[a] IAC was inhibited even after re-extraction, therefore the sample was excluded from the study.
[b] IAC was inhibited initially, but valid after re-extraction.
